# Osteoclasts and osteoarthritis: Novel intervention targets and therapeutic potentials during aging

**DOI:** 10.1111/acel.14092

**Published:** 2024-01-29

**Authors:** Haojue Wang, Tao Yuan, Yi Wang, Changxing Liu, Dengju Li, Ziqing Li, Shui Sun

**Affiliations:** ^1^ Department of Joint Surgery, Shandong Provincial Hospital, Cheeloo College of Medicine Shandong University Jinan Shandong China; ^2^ Department of Joint Surgery Shandong Provincial Hospital Affiliated to Shandong First Medical University Jinan Shandong China; ^3^ Orthopaedic Research Laboratory, Medical Science and Technology Innovation Center Shandong First Medical University and Shandong Academy of Medical Sciences Jinan Shandong China

**Keywords:** aging, bone resorption, osteoarthritis, osteoclast, subchondral bone

## Abstract

Osteoarthritis (OA), a chronic degenerative joint disease, is highly prevalent among the aging population, and often leads to joint pain, disability, and a diminished quality of life. Although considerable research has been conducted, the precise molecular mechanisms propelling OA pathogenesis continue to be elusive, thereby impeding the development of effective therapeutics. Notably, recent studies have revealed subchondral bone lesions precede cartilage degeneration in the early stage of OA. This development is marked by escalated osteoclast‐mediated bone resorption, subsequent imbalances in bone metabolism, accelerated bone turnover, and a decrease in bone volume, thereby contributing significantly to the pathological changes. While the role of aging hallmarks in OA has been extensively elucidated from the perspective of chondrocytes, their connection with osteoclasts is not yet fully understood. There is compelling evidence to suggest that age‐related abnormalities such as epigenetic alterations, proteostasis network disruption, cellular senescence, and mitochondrial dysfunction, can stimulate osteoclast activity. This review intends to systematically discuss how aging hallmarks contribute to OA pathogenesis, placing particular emphasis on the age‐induced shifts in osteoclast activity. It also aims to stimulate future studies probing into the pathological mechanisms and therapeutic approaches targeting osteoclasts in OA during aging.

## Graphical abstract

Osteoclast serves as a pivotal mediator and communication hub within the subchondral bone, accelerating the onset and progression of osteoarthritis (OA). Aging‐related alterations (cellular senescence, inflammaging, mitochondrial dysfunction, abnormal histone modification, non‐coding RNAs, DNA methylation, dysregulated autophagy and molecular chaperones) significantly enhance osteoclast activities, thereby contributing to OA pathogenesis.

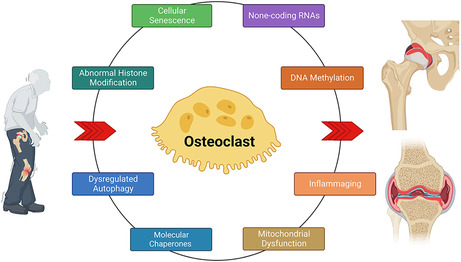

**Abbreviations**
acetyl‐CoA: acetyl coenzyme A
ACLT: anterior cruciate ligament transection
ACLY: ATP‐citrate lyase
AGEs: advanced glycation end products
Atg: autophagy‐related genes
BMU: basic multicellular unit
BRC: bone remodeling compartment
CAR: CXCL12‐abundant reticular
circRNAs: circular RNAs
CPGIs: CpG islands
c‐Src: proto‐oncogene tyrosine‐protein kinase
CXCL10: C‐X‐C motif ligand 10
CXCL12: CXC chemokine ligand 12
DAMPs: damaged‐associated molecular patterns
DCC: deleted in colorectal cancer
DEGs: differentially expressed genes
DNMTs: DNA methyltransferases
DOT1L: disruptor of telomeric silencing 1‐like
DRG: dorsal root ganglion
ER: endoplasmic reticulum
Ero1: endoplasmic reticulum oxidoreductin 1
EVs: extracellular vesicles
FDA: Food and Drug Administration
FOXOs: forkhead box proteins
GM‐CSF: granulocyte‐macrophage colony‐stimulating factor
H3K9Ac: H3 lysine 9 acetylation
HDACs: histone deacetylases
HIF: hypoxia‐inducible factor
HO‐1: heme oxygenase‐1
HSCs: hematopoietic stem cells
Hsp: heat shock protein
ITAM: immunoreceptor tyrosine‐based activation motif
Jmjd3: jumonji domain‐containing 3
KDM: histone lysine demethylase
KLFs: Kruppel‐like factors
lncRNAs: long non‐coding RNAs
MALPs: bone marrow adipogenic lineage precursors
MIA: monoiodoacetate
miRNAs: microRNAs
MITF: microphthalmia transcription factor
MnSOD: manganese superoxide dismutase
MRI: magnetic resonance imaging
mtDNA: mitochondrial DNA
NADPH: nicotinamide adenine dinucleotide phosphate
NEAT1: nuclear‐enriched abundant transcript 1
NFATc1: nuclear factor of activated T cells cytoplasmic 1
NGF: nerve growth factor
Nox1: nicotinamide adenine dinucleotide phosphate oxidase 1
NSAIDs: nonsteroidal Anti‐inflammatory Drugs
nts: nucleotides
OA: osteoarthritis
OSCAR: osteoclast‐associated receptor
OXPHOS: oxidative phosphorylation
P2RXs: purinergic ionotropic receptors
PDI: protein disulfide isomerase
PRRs: pathogen‐recognition receptors
ROS: reactive oxygen species
RyR: ryanodine receptor
SAM: S‐adenosyl‐L‐methionine
SAM: S‐adenosylmethionine
SASP: senescence‐associated secretory phenotype
SAβ‐gal: senescence‐associated β‐galactosidase
SCAPs: senescent cell antiapoptotic pathways
SERCA: sarco/endoplasmic reticulum Ca^2+^ ATPase
siRNA: small interfering RNA
SIRT: sirtuin
sRAGE: soluble receptor for advanced glycation end product
TF: transcription factor
TLR: toll‐like receptor
TNF‐α: tumor necrosis factor‐α
TRAF6: TNF receptor‐associated factor 6
TRP: transient receptor potential
TSS: transcription start sites
UPR: unfolded protein reaction
VGCC: voltage‐gated Ca^2+^ channel
μCT: micro–computed tomographic

## 1 INTRODUCTION

Osteoarthritis (OA) is a degenerative disease that entails the whole joint including synovium, cartilage, subchondral bone, and other periarticular tissues (Ramos‐Mucci et al., 2020). The development and integration of OA prevalence studies are largely affected by discrepancies in disease definitions, age groups, and gender populations (Pereira et al., 2011). It is estimated, however, more than 500 million people suffer from OA worldwide, wherein at least 70% of them are over 55 years old, bringing substantial medical burdens, especially in countries with the high Socio‐Demographic Index (Lo et al., 2021; Global Burden of Disease Collaborative Network, 2022). For instance, in the United States, OA affects 13.4% of the population (Cisternas et al., 2016), producing $373.2 billion direct costs and $113.2 billion indirect costs annually (Hochberg et al., 2020). The etiology of OA is heterogeneous and blurry. Many risk factors such as obesity, local injury, and female gender are related, but among which, aging is one of the most prominent causes that has been widely accepted to contribute to OA, even if the exact mechanism between OA and aging is still unclear (Sanchez‐Lopez et al., 2022). Besides, aging is a high alloplastic process differing in various populations, and the vague mechanisms of aging in the pathophysiology of OA bring about quite a few difficulties in the treatment of this typical geriatric disease.

Current treatment of OA can be described as a stepped therapy, including non‐pharmacological management, pharmacotherapy treatment, and surgical interventions. However, limitations, to some extent, exist in these approaches. Although exercise therapy as the first line treatment of OA has been recommended in clinical guidelines, it is difficult to prescribe a personalized and exact dose of exercise for patients in clinical practice, and the actual effectiveness highly relies on the cooperation of the individual patient (NICE, 2022). Nonsteroidal Anti‐inflammatory Drugs (NSAIDs) are the most efficient pharmacotherapy to relieve OA pain (da Costa et al., 2021). However, considering the demand for long‐term intake and subsequential side effects such as gastrointestinal cardiovascular, and renal detriment, the period and safety of continuous use of NSAIDs is still controversial (Magni et al., 2021). On the other hand, NSAIDs can not always provide a continuous curative effect on OA pain (Zhu et al., 2019). When nonsurgical approaches fail, joint arthroplasty is generally the most effective but also the last means for advanced OA. The limited lifespan of artificial prosthesis makes it hard to be accepted by younger patients, and surgical risks and complications result in hesitation in older patients with frailty (Cook et al., 2022; Delanois et al., 2017). Therefore, the limitations in current approaches urge the development of novel therapeutics in order to effectively delay the progression of OA and relieve clinical symptoms, which largely require a better understanding of disease pathogenesis.

Early studies of OA pathogenesis pay more attention to dealing with the unrecoverable cartilage and chondrocytes, and relevant medications such as hyaluronans, glucosamine, as well as chondroitin are developed (Dieppe & Lohmander, 2005; Towheed et al., 2005). However, the efficacy is still under huge controversy (Fransen et al., 2015; Richette et al., 2015; Yang et al., 2015), indicating the underdevelopment of the early understanding of pathogenesis. As the most potential risk factor for OA onset and progression, aging affects a large quantity of the OA population by mediating pathological alterations in the whole synovial joint (Motta et al., 2023; Rahmati et al., 2017). Compelling evidence exists that senescence‐associated variations in chondrocytes, osteoclasts, osteoblasts, synovial cells, and the joint microenvironment have been implicated in the pathogenesis and progression of OA (Wu, Liu, et al., 2022). To reflect at the pathological level, cartilage degeneration and subchondral bone deterioration are characteristic changes of OA (Li, Roemer, et al., 2023). For which, the acceleration and interruption of bone turnover result in trabecular bone loss, sclerosis, and osteophyte formation in subchondral bone, and the happening of subsequent cartilage damage and erosion as well as other clinical symptoms for example OA pain and functional limitation is at least partly attributed to subchondral bone alterations (Driban et al., 2011; Yusuf et al., 2011). Functioning as the bone‐resorbing machine, osteoclast plays a crucial role in mediating subchondral bone metabolism and turnover, thus contributing to part of OA pathological changes (Hu et al., 2021; Veis & O'Brien, 2023). It is also important to note that osteoclast activities are largely affected by the aging process (Sun et al., 2021). Given the intimate association between hallmarks of aging (epigenetic changes, proteostasis networks, cellular senescence, and mitochondrial dysfunction) and osteoclast biology (Ling et al., 2021; Marini et al., 2016; Pimenta‐Lopes et al., 2023; Wang, Kong, et al., 2023), mechanism studies of OA based on this pointcut have attracted considerable attention recently.

Therefore, in this review, we will elucidate the role of osteoclast in OA pathogenesis from molecular biological aspects to clinical symptom settings, with a particular focus on the alternations under the aging context. Moreover, the hallmarks of aging that are involved in OA pathogenesis and osteoclast will be elaborated in the following content, and the promising therapeutics targeting these hallmarks will also be reviewed and discussed.

## 2 THE SIGNIFICANT ROLE OF OSTEOCLASTS IN BONE REMODELING AND OA

### 2.1 How do osteoclasts maintain bone homeostasis

#### 2.1.1 Physiological osteoclast biology

Bone remodeling is a coupled process mediated by two primary bone cells within the basic multicellular unit (BMU), which are the bone resorption osteoclasts and the bone formation osteoblasts. The continuing and balancing of this dynamic process is required for bone turnover and homeostasis maintenance (Feng & McDonald, 2011). Osteoclasts, originating from hematopoietic stem cells (HSCs) /myeloid cells /bone marrow monocytes axis, process the ability to degrade old and damaged bone. Besides canonical hematopoietic origin, brand‐new bone cells named osteomorphs were recently found by McDonald et al. (2021), which come from osteoclasts fission and exhibit distinct morphological and transcriptional properties than traditional osteoclasts. The mature osteoclasts that derived from osteomorphs fusion were also identified to possess bone‐resorbing functions (McDonald et al., 2021).

#### 2.1.2 Communication between osteoclasts and other bone cells during bone remodeling

The process of bone remodeling usually sees bone resorption precede bone formation and requires robust intercellular communication, particularly between osteoclasts and osteoblasts, in the bone remodeling compartment (BRC) (Bolamperti et al., 2022; Parfitt, 1994). BRC provides a finite space for these intercellular communications to support the sophisticated bone remodeling process (Chim et al., 2013). Aging, however, disrupts these programmed processes within the BRC, leading to phenomena such as local inflammation, impaired osteoblast function, and increased osteoclast activity (Wang, Yang, et al., 2020). As one of the important communicating hubs, osteoclast is constantly influenced by the bone microenvironment and interacts with adjacent cells, modulating their functions accordingly. Notably, osteoclastogenic signaling pathways are regulated by the RANKL/RANK/OPG axis, while the calcium signaling pathway will potentiate this process if triggered by costimulatory factors (Wang, Yang, et al., 2020).

Due to previous extensive studies on the RANKL signaling pathway, this review will not discuss on this topic further (Novack & Teitelbaum, 2008; Veis & O'Brien, 2023). RANKL abundance predominantly impacts osteoclast differentiation and function, even though OPG, the soluble decoy receptor for RANKL without membrane‐bound potential, can competitively inhibit the RANKL‐RANK binding (Han et al., 2018; Veis & O'Brien, 2023). Therefore, affecting cells contributing to RANKL supply directly influences osteoclast survival and functional activity. So far, various cell types have demonstrated the capacity to produce RANKL and instigate osteoclastogenesis under specific physiological or pathological conditions (Gruber, 2019; Veis & O'Brien, 2023). Research progresses, inclusive of our recent work, suggest potential RANKL sources such as bone marrow adipogenic lineage precursors (MALPs), CXC chemokine ligand 12 (CXCL12)‐abundant reticular (CAR) cells, lymphocytes, synovial fibroblasts, and periodontal ligament cells (Kong et al., 1999; Sato et al., 2006; Tsukasaki & Takayanagi, 2019; Veis & O'Brien, 2023; Yu et al., 2021). While direct contact between osteoblast and osteoclast is the major pathway for the membrane‐bounded RANKL to conjugate with RANK (Martin & Sims, 2015), other membrane‐bound factors of EphB4 and FASL in osteoblast can also display inhibitory effects on osteoclast thus displaying bidirectional effects (Wang, Liu, et al., 2015; Zhao et al., 2006). Aside from direct contact, secreted proteins facilitate osteoblast–osteoclast communication (Han et al., 2018). Examples include WNT5a, WNT16, SEMA3A, SOST, and VEGFA, all secreted by osteoblast lineage cells (Han et al., 2018). Conversely, coupling factors like TGF‐β and IGF‐1 that released from the bone matrix or BMPs and ephrinB2 that directly expressed by osteoclasts could promote osteogenesis and initiate the reversal phase during bone remodeling (Bolamperti et al., 2022; Bordukalo‐Niksic et al., 2022; Zhao et al., 2006).

Located adjacent to the subchondral bone, chondrocytes, which are key cellular components of articular cartilage and epiphysis, have been shown to interact with osteoclasts. In *β‐catenin* CKO mice, there is observed decreased OPG expression and increased RANKL expression in chondrocytes, alongside enhanced osteoclastogenesis, indicating the crucial role of the *β‐catenin* signaling pathway in chondrocytes for osteoclast regulation (Kwan Tat et al., 2009; Wang et al., 2014). In addition, the activation of the p38 signaling pathway in apoptotic chondrocytes potentially facilitates CX3CL1 expression and secretion, resulting in the recruitment of osteoclast precursors via chemotaxis (Guo et al., 2022). The osteoclast‐associated receptor (OSCAR), expressed by osteoclast lineage cells, can be triggered by chondrocytes‐induced collagen, hence aiding in the formation and function of osteoclasts (Park et al., 2020). An elevation in OSCAR expression was recognized in OA cartilage, particularly in damaged regions, and inhibiting or reducing OSCAR could mitigate both the damage to cartilage and the loss of subchondral bone in OA (Park et al., 2020).

#### 2.1.3 Aging exerts pluralism regulatory pathways on osteoclast behaviors

Classical age‐related changes of skeleton involve the decline of both cancellous bone mass and cortical bone thickness, as well as increased intracortical porosity. These alterations result from heightened osteoclast bone resorption activity and an imbalance in the bone remodeling process (Piemontese et al., 2017; Ucer et al., 2017), which have also been shown to strongly correlate with OA development and symptoms (Hu et al., 2021). Therefore, given the crucial role of aging in the regulation of osteoclast activities and its significance as a causal factor in OA pathogenesis, the impact of aging on osteoclast behavior is non‐negligible in OA study.

The RANKL/RANK/OPG axis continues to dominate osteoclast behavior changes during the aging process. It has been well‐documented that excessive RANKL and M‐CSF expression, along with a decrease in OPG levels, can be identified in the entire bone and bone marrow cells (Cao et al., 2005; Chung et al., 2014; Makhluf et al., 2000). The upregulation of Gata4, a senescence‐associated transcription factor, can lead to an increased Tnfrsf11 (encoding RANKL) mRNA level in osteocytes (Kim et al., 2020). Conversely, the downregulation of Piezo1, a crucial mediator for bone homeostasis and mechanotransduction, results in decreased Tnfrsf11b (encoding OPG) expression (Li, Zhang, et al., 2023). Additionally, aging‐related alterations also reprogram osteoclast and their precursor cells, leading to a more aggressive behavior. A possible contributing factor could be a switch from lymphoid to myeloid cells in the hematopoietic lineage, leading to the expansion of the osteoclast precursor pool (Ding et al., 2022). Changes in the methylation status of the TM7SF4 and CTSK gene, along with increased expression of RANK, c‐fms, TM7SF4 and CTSK, could further foster the osteoclastic ability of precursor cells (Cao et al., 2005; Moller et al., 2020).

Beyond the canonical RANKL/RANK/OPG axis, increased expressions of sirtuin (SIRT) 3 and hypoxia‐inducible factor (HIF) − 2α, as exhibited by osteoclast lineage or bone, have been found to correlate with age‐related osteoclastogenesis (Ho et al., 2017; Lee et al., 2021). Concurrently, the noticeable rise in toll‐like receptor (TLR) 9 gene expression coupled with a decline in TLR4 gene expressions during aging greatly intensifies the cellular responsiveness to the imflammaging milieu in osteoclasts, resulting in a surge in the expression of inflammatory mediators and damaged‐associated molecular patterns (DAMPs) (Akkaoui et al., 2021; Albuquerque‐Souza et al., 2022). Additionally, the excessive release of TGFβ1 from resorbed bone during aging can incite the ubiquitination degradation of TRAF3 in mesenchymal progenitor cells. This process promotes RANKL expression by activating NF‐κB RelA and RelB, while simultaneously inhibiting osteoblast formation through the GSK3β/β‐catenin signaling pathway (Li et al., 2019). The degradation of TRAF3 in osteoclast also facilitates the activation of NF‐κB‐inducing kinase, fostering the formation of the p52/RelB heterodimer and thereby promoting osteoclast differentiation and function (Boyce et al., 2018).

**FIGURE 1:**
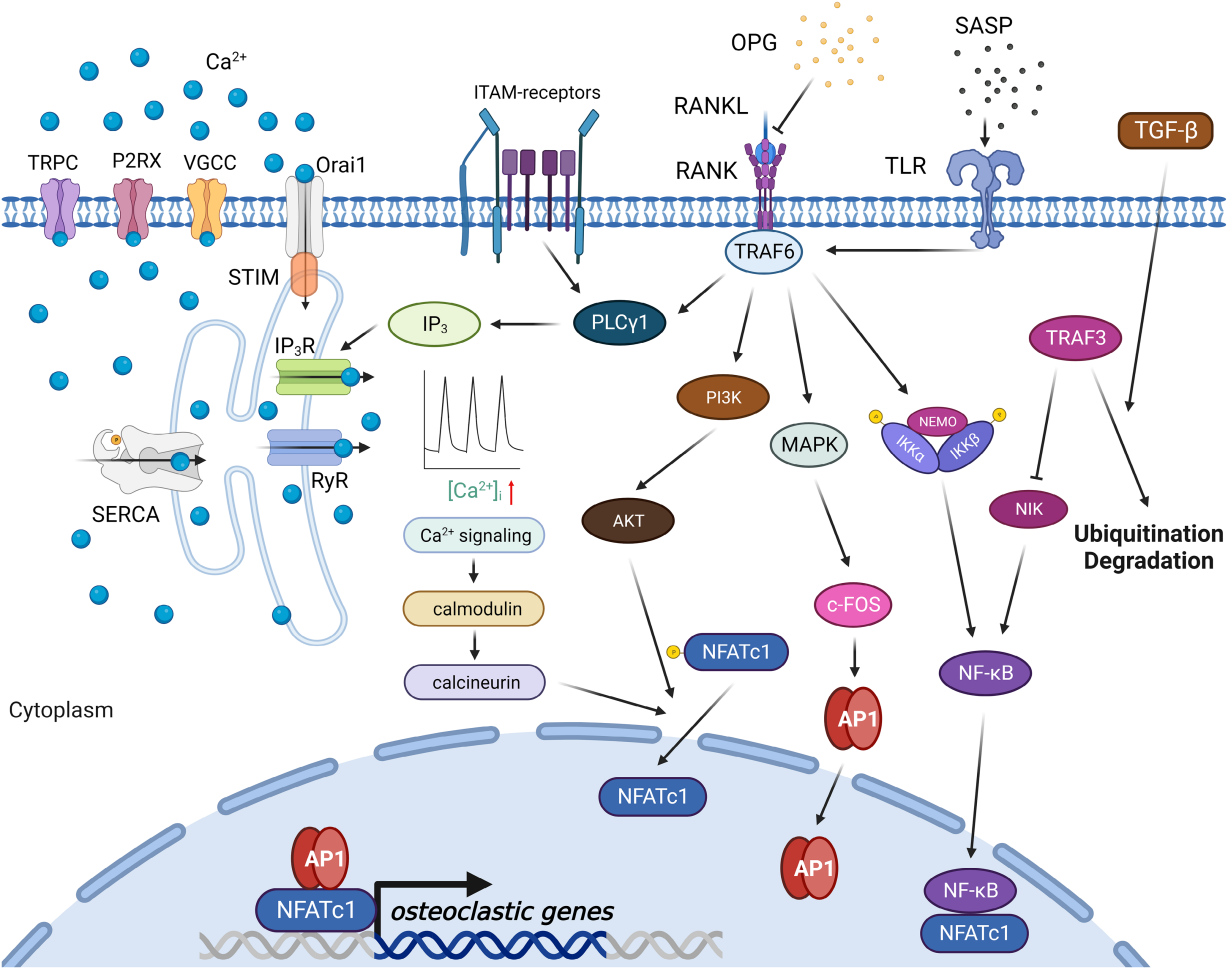
Sophisticated osteoclast regulatory network in physiological and aging condition. The osteoclasogenesis is governed by numerous signaling pathways. The binding of RANKL to RANK facilitates the recruitment of TRAF6, which then triggers various downstream signaling pathways, including NF‐κB, MAPK, PI3K‐AKT, and the calcium signaling pathway. These pathways, in turn, influence transcription factors like NFATc1 and AP‐1, subsequently promoting the expression of genes related to osteoclastogenesis. AP1, activator protein‐1; IKKα, inhibitor of NF‐κB kinase subunit α; IKKβ, inhibitor of NF‐κB kinase subunit β; ITAM, immunoreceptor tyrosine‐based activation motif; MAPK, mitogen‐activated protein kinases; NEMO, NF‐κB essential modulator; NFATc1, nuclear factor of activated T cells 1; NF‐κB, nuclear factor kappa B; NIK, NF‐κB‐inducing kinase; OPG, osteoprotegerin; P2XR, purinergic ionotropic receptor; PI3K, phosphoinositide 3‐kinase; RANK, receptor activation of NF‐κB; RANKL, receptor activation of NF‐κB ligand; RyR, ryanodine receptor; SASP, senescence‐associated secretory phenotype; SERCA, Sarco/endoplasmic reticulum Ca^2+^ ATPase; TLR, toll‐like receptor; TRAF3, tumor necrosis factor receptor‐associated factor 3; TRAF6, tumor necrosis factor receptor‐associated factor 6; TRPC, transient receptor potential channel; VGCC, voltage‐gated Ca^2+^ channel.

Osteoclast behaviors are also predominantly influenced by the calcium oscillation and associated signaling pathways during aging (Kang et al., 2020; Martin & Bernard, 2018). The initiation of calcium oscillation through the PLCγ/IP_3_ axis, following upstream RANK or immunoreceptor tyrosine‐based activation motif (ITAM) receptor activation, facilitates calcineurin which functions as a phosphatase to promote nuclear factor of activated T cells cytoplasmic (NFATc) 1 (NFATc1) dephosphorylation and nuclear translocation (Hirotani et al., 2004; Koga et al., 2004; Negishi‐Koga & Takayanagi, 2009; Son et al., 2012). The calcium oscillation is finely tuned by two pathways: (1) by calcium channel proteins on plasma membrane, including nonselective transient receptor potential (TRP) channels, purinergic ionotropic receptors (P2RXs), voltage‐gated Ca^2+^ channel (VGCC), and Orai 1; (2) by calcium channel or pump proteins found in the endoplasmic reticulum (ER) or other organelles, like the IP_3_ receptor, Sarco/ER Ca^2+^ ATPase (SERCA), and ryanodine receptor (RyR) (Kang et al., 2020; Okada et al., 2020). Previous research has demonstrated an elevation of calcium levels in senescent cells, attributable to either calcium influx via the plasma membrane or calcium release from the ER (Huang et al., 2022; Martin et al., 2023; Toescu et al., 2004). Such an increase might further exacerbate senescent phenotypes. For example, the calcium/calcineurin/NFATc1 axis can also promote type 2 IP_3_ receptor expression to strengthen ER Ca^2+^ release, or suppress ATF expression to activate p53‐dependent cellular senescence (Sankar et al., 2014; Wu et al., 2010) (Figure 1). Therefore, as a key effector in subchondral bone, osteoclasts may be involved in the pathophysiological changes of OA, contributing to clinical symptoms such as OA pain, joint stiffness, and functional limitation.

### 2.2 Subchondral bone osteoclasts are major mediators of OA characteristic symptom

#### 2.2.1 What is OA pain

Pain is the most prominent clinical symptom of OA and the primary cause of patients asking for medical assistance. Structure alterations in subchondral bone, articular cartilage, and other soft tissues inside the joint can all be potential sources of OA pain. However, it is also proved that pain grade may not coincide with the radiographic manifestation (Bacon et al., 2020). The emergence of OA pain can also be attributed to acidic substances released by osteoclasts, sensory nerve innervation, bone and cartilage destruction, and senescence‐associated secretory phenotype (SASP). Therefore, OA pain in patients extremely differs in various subtypes and disease stages. Although current pharmacotherapy for OA patients in early or advanced stages claims to relieve joint pain, commonly used medicines like NSAIDs and analgesics may not always be effective in clinical practice (Weng et al., 2023). Still, a deeper investigation of OA pathogenesis will still be significantly meaningful for OA treatment and symptom relief.

Aging has been pinpointed as a pivotal influence in the development of OA pain. In 20‐month‐old mice that have not undergone additional intervention, mild OA has been identified in the knee joint, exacerbating knee hyperalgesia and mechanical allodynia (Geraghty et al., 2023). Age‐related alterations in the sensory aspect of the pain modulatory network, along with the variations in cellular populations of the dorsal root ganglion (DRG), could have a significant relationship with chronic OA pain (Da Silva et al., 2021; Geraghty et al., 2023). Additionally, the overproduction of Netrin‐1 by elevated osteoclasts during the aging process also facilitates sensory innervation, thereby escalating pain sensitivity (Ni et al., 2019). The comprehensive involvement of hyperactivated osteoclasts may be a major contributor to OA pain, a topic we will elucidate in more detail later.

#### 2.2.2 Potential roles of osteoclasts in OA pain during disease progression

Enhanced osteoclastogenesis and bone resorption are prominent characteristics of OA in the early stage (Siebelt et al., 2014; Wu, Yuan, et al., 2022). Acidification of the resorption pit induced by proton‐secreting osteoclasts is essential for the degradation of inorganic bone matrix (Teitelbaum, 2000). However, exceeding acidification may stimulate nociceptive nerves in the subchondral bone, resulting in bone‐originated pain (Yoneda et al., 2015). In addition to continuous nociceptive signals from the cartilage and subchondral bone, generally appearing with angiogenesis and sensory innervation are also typical pathological changes in the early stage of OA, thereby providing another source of OA pain caused by the peripheral nerve sensitization due to innervation of the sensory nerve (Thakur et al., 2014). Aging affects these processes greatly, not only facilitating osteoclast cellular activities, but also contributing to the inflammatory microenvironment. For instance, sensory innervation and neovascularization can be identified in the degenerated joint, which is characterized by an increased expression of inflammatory factors (Kim, Ali, et al., 2015). This phenomenon could be linked to the heightened levels of nerve growth factor (NGF) and VEGF that typically occur under conditions of aging and inflammation (Gatsiou et al., 2021; Gugliandolo et al., 2018; Mahdee et al., 2019; Marazita et al., 2016).

Increased nerve fibers are shown to be agreeing with excessive osteoclastogenesis and the formation of type H vessels (Su et al., 2020). Furthermore, a direct influence of osteoclasts on nerve growth has been demonstrated. Within 1 week of administering the surgery of anterior cruciate ligament transection (ACLT) in mice, Netrin‐1, which is secreted by osteoclasts, interacts with the deleted in colorectal cancer (DCC) receptor and significantly enhances sensory innervation and OA pain. In contrast, noticeable cartilage degradation narrowly occurred 4 weeks postoperatively (Zhu et al., 2019). Alleviation of innervation and pain symptoms were observed in the Netrin‐1 or Dcc knockdown models or when osteoclast activity was inhibited by alendronate (Zhu et al., 2019). Interestingly, sensory innervation was not found in the subchondral bone until 28 days post monoiodoacetate (MIA)‐induced OA (Morgan et al., 2022). Therefore, joint destabilization and mechanical force disturbance, rather than the inflammatory condition of the joint space, may be essential for osteoclast‐induced sensory innervation in the subchondral bone and articular cartilage during early‐stage OA.

**FIGURE 2:**
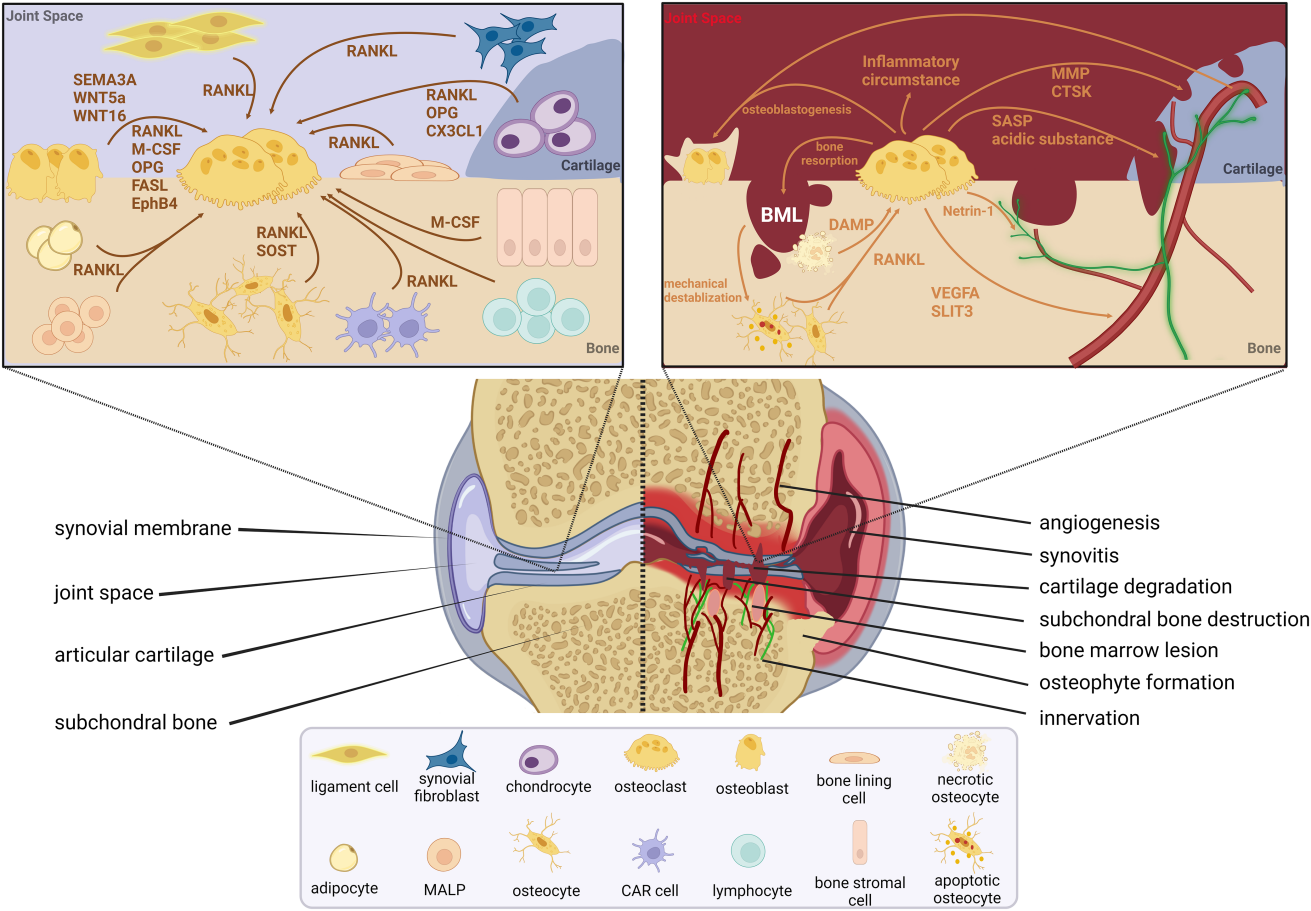
Role of osteoclasts in physiological and pathological conditions. Subchondral osteoclasts serve as a communication hub and extensively participate in physiological bone homeostasis and pathological bone alterations. In physiological conditions, bone homeostasis is maintained through a delicate balance of bone formation and resorption, wherein osteoclasts play an indispensable role in bone resorption and are affected by diverse factors secreted from neighboring cells. In pathological conditions, such as OA, necrotic and apoptotic osteocytes and neighboring cells in the bone marrow lesion (BML) area cause the overactivation of osteoclasts, subsequently resulting in a cascade of events that contribute to disease progression, including increased angiogenesis, sensory innervation, cartilage and bone destruction, and osteophyte formation. BML, bone marrow lesion; CTSK, cathepsin K; CX3CL1, C‐X3‐C motif chemokine ligand 1; DAMP, damaged‐associated molecular pattern; EphB4, ephrin type‐B receptor 4; FASL, Fas ligand; M‐CSF, monocyte/macrophage colony‐stimulating factor; MMP, matrix metalloproteinase; OPG, osteoprotegerin; PDGF‐BB, platelet‐derived growth factor‐BB; RANKL, receptor activation of NF‐κB ligand; SASP, senescence‐associated secretory phenotype; SLIT3, slit guidance ligand 3; SOST, sclerostin; VEGFA, vascular endothelial growth factor A; WNT16, WNT family member 16; WNT5a, WNT family member 5A.

In addition to hyperalgesia resulting from sensory innervation, increased mechanical and chemical stimuli to subchondral nerves due to BML also contribute to OA pain. Coinciding with OA progression, BML is a typical radiographic feature highly associated with excessive osteoclast activity and accelerated bone remodeling, and the occurrence of this structural abnormality has been found to correlate with aging (Driban et al., 2011; Guermazi et al., 2012). Abnormal mechanical loading and inflammatory condition arising from BML could potentially irritate nociceptors within the bone, and neuropathic pain may be activated by the direct damage of BML to nerve fibers (Ebbinghaus et al., 2019; Kuttapitiya et al., 2017; Nencini & Ivanusic, 2017). Substantial evidence demonstrates a strong correlation between the size of BML as visualized on magnetic resonance imaging (MRI) and the intensity of pain experienced by patients (Yusuf et al., 2011). Pathologically, BML exacerbates the destruction of superficial articular cartilage and causes mechanical destabilization, which makes cartilage and subchondral bone in the medial tibial plateau experience more severe wear and tear, further promoting joint imbalance and creating a vicious cycle of BML, cartilage destruction, and mechanical destabilization (Guermazi et al., 2012). Intense pain occurs when deeper erosion happens to the cartilage and subchondral bone, exposing the nociceptors within the innervated subchondral bone and even the damaged cartilage directly to inflammatory factors and chemical substances that are released into the joint space. Interestingly, by inhibiting osteoclast activity, bisphosphate treatment has demonstrated promising therapeutic effects on both BML and pain (Laslett et al., 2012), indicating the important role of osteoclasts during the pathological process (Figure 2).

## 3 HOW DO HALLMARKS OF AGING PARTICIPATE IN THE PATHOGENESIS OF OA THROUGH OSTEOCLASTS

OA is a prevalent chronic degenerative joint disease closely related to aging. Therefore, understanding how aging contributes to OA progression from a molecular biological perspective is crucial in deciphering the disease's pathology. Nine common hallmarks of aging have been identified in various senescent species, tissues and cells: mitochondrial dysfunction, epigenetic changes, loss of proteostasis, cellular senescence, stem cell exhaustion, altered intercellular communication, genome instability, telomere attrition, and deregulated nutrient‐sensing (Lopez‐Otin et al., 2013). These biomarkers have been extensively employed in the studies of numerous degenerative diseases, with some have been found to directly or indirectly impact the aging osteoclasts (Hou et al., 2019). Hence, this section utilizes the hallmarks of aging as a bridge and guide for delving deeper into the relationship between osteoclastic alterations and the aging context, as well as their roles in OA pathogenesis.

### 3.1 Epigenetics

Epigenetics refers to alterations in gene expression that occur without changing the underlying inherent sequence, including DNA methylation, histone modifications and noncoding RNAs (Husain & Jeffries, 2017). While numerous studies have investigated epigenetic alterations in OA, few have focused on osteoclasts. Indeed, significant changes in gene expression are notified in osteoclasts during OA progression. The hyperactivity of osteoclasts in early‐stage OA and functional recession of osteoclasts in advanced‐stage OA may be attributed to changes in gene expression resulting from epigenetic alterations (Burr & Gallant, 2012). Therefore, addressing these abnormal epigenetic alterations associated with osteoclasts may restore ordinary bone resorption and contribute to effective OA treatment.

#### 3.1.1 DNA methylation

DNA methylation typically refers to the transfer of a methyl group from S‐adenosylmethionine (SAM) to DNA through the action of DNA methyltransferases (DNMTs) (Li, Sun, et al., 2021), which generally happens on the 5‐position carbon of cytosines in CpG dinucleotides (Soto‐Palma et al., 2022). CpG islands (CGIs) are regions with CpG‐rich DNA sequences, usually located within or near gene promotors (Bird, 1986; Bird et al., 1985; Saxonov et al., 2006), and methylation of CGIS promotors could silence gene expression (Robertson & Wolffe, 2000). Numerous signature genes with abnormal methylation status have been identified in OA patients (Cai et al., 2022). Notably, the differentiation of monocytes into osteoclasts is also influenced by both demethylation and methylation processes. PU.1 is a key mediator in this process, as it can recruit DNMT3b or TET2 to its target genes to induce either hypermethylation or hypomethylation of these genes (de la Rica et al., 2013). CTSK and TM7SF4 are typically hypomethylated genes involved in osteoclastogenesis, especially following the stimulation of RANKL and M‐CSF (de la Rica et al., 2013). While osteoclasts differentiated from CD14‐positive monocytes of aging donors exhibit enhanced bone resorption activity, an in vitro study confirmed that changes in DNA methylation status were associated with osteoclasts formation and function, wherein hypomethylation of the promotor domains of *TM7SF4* and *CTSK* genes was notably observed in aging population, promoting gene expression and leading to increased fusion abilities and aggressive bone resorption (Moller et al., 2020). Thus, the hypomethylation‐activated characteristic genes of osteoclasts during aging may be correlated with OA pathogenesis.

#### 3.1.2 Histone post‐translational modifications

Histone modifications, including methylation, demethylation, acetylation, deacetylation, and many other modifications, involve altering residues in histone tails and are intensively associated with transcription factor (TF) binding, thereby affecting gene expression (Lawrence et al., 2016). Numerous studies have explored changes in methylation and acetylation status in OA, providing a comprehensive understanding of their roles in OA chondrocytes (Wan et al., 2021). However, few studies have attempted to link histone modifications in osteoclasts to OA.

Histone deacetylation, typically associated with the suppression of promotors or enhancers, is among the most well‐studied aspects of osteoclasts (Astleford et al., 2020; Bradley et al., 2015). Histone deacetylases (HDACs), which remove acetyl groups from histones, have been proven to regulate osteoclast formation and function (Bradley et al., 2015). Eighteen distinct enzymes that belong to the HDACs family have been divided into four classes, exerting pleiotropic effects on osteoclasts (Bradley et al., 2015; Vrtacnik et al., 2014). SIRTs are a family of NAD^+^‐dependent deacetylases belonging to class III HDACs and possess anti‐aging potential (Lopez‐Otin et al., 2013). Importantly, their association with OA has been recently reported (He et al., 2020). SIRT1 acts as a suppressor of the NF‐κB signaling pathway, and its conditional deletion in osteoclasts leads to enhanced osteoclastogenesis and bone resorption (Edwards et al., 2013). Forkhead box proteins (FOXOs) transcription factors, which inhibit osteoclastogenesis, have been found as targets of SIRT1 in osteoclasts, revealing an unusual HDACs‐mediated promotion of gene expression via the deacetylation of FOXOs‐target genes (Kim, Han, et al., 2015). By using bone marrow‐derived monocytes, Wei Zhang et al. proved that a SIRT1 agonist could inhibit osteoclastogenesis. As a pivotal downstream TF of the RANKL‐RANK signaling in osteoclasts, the NFATc1 along with c‐Fos were found to be downregulated due to increased SIRT1 expression and deacetylated FOXO1 (Zhang et al., 2023). In addition to SIRT1, SIRT6 could also inhibit osteoclast formation and bone resorption (Zhang et al., 2016). Through inducing osteoclastogenesis from both young and aged mice, Moon et al. observed lower SIRT6 expression in aged osteoclasts. Further experiments found that SIRT6‐mediated deacetylation of ERα protein could increase its stability and subsequently promote osteoclast apoptosis via the transcription of Fas ligand (Moon et al., 2019). Thus, the SIRT family can regulate osteoclast formation and function in both histone and non‐histone manner. Various HDACs from class I and class II are also intimately associated with osteoclasts. For instance, the increased expression of HDAC5 and HDAC8 was observed during osteoclastogenesis (Cantley et al., 2011). Moreover, the downregulation of osteoclast‐specific genes, as well as inhibition of osteoclast differentiation and bone resorption were found when HDAC6 was knocked down or HDAC1 and HDAC2 were inhibited (Algate et al., 2020; Wang et al., 2018).

In contrast to the suppressive effect of deacetylation on gene expression, histone acetyltransferases tend to promote gene expression by inducing histone acetylation (Neganova et al., 2022). Produced by ATP‐citrate lyase (ACLY), acetyl coenzyme A (acetyl‐CoA) serves as the major donor of the acetyl group necessary for histone acetylation (Icard et al., 2020). Increased ACLY expression and activation have been caught in an IL‐1β‐induced OA model, promoting the expression of iNOS, MMP3, and MMP13 via the acetylation of H3K9 and H3K27 (Chen et al., 2018). Moreover, either pharmacological inhibition or knockdown of ACLY in osteoclasts was found to suppress osteoclastogenesis and bone resorption (Guo, Kang, et al., 2021). In terms of mechanism, following RANKL stimulation, ACLY translocates from the cytoplasm to the nuclear, promoting the acetylation of H3K9, H3K27, H3K14, and H3K18 with the assistance of GCN acetyltransferase (Guo, Kang, et al., 2021). By further mediating the PI3K‐AKT signaling pathway, Rac1, a crucial factor in managing the formation of F‐actin ring and the function of bone resorption in osteoclasts, is regulated by ACLY (Guo, Kang, et al., 2021). In addition to ACLY, Kruppel‐like factors (KLFs) are TFs that regulate cellular differentiation and activate monocytes, and knockdown of Klf2 in osteoclasts increased histone H3 lysine 9 acetylation (H3K9Ac) and H4K8Ac in the promoter region of Beclin1, leading to Beclin1 overexpression and inhibition of osteoclastogenesis (Laha et al., 2019).

Histone methylation refers to the transfer of up to three methyl groups from the common methyl donor S‐adenosyl‐L‐methionine (SAM) to lysine or arginine residues of histones (Horiuchi et al., 2013; Rungratanawanich et al., 2023). Histone methyltransferases can be generally classified into three different classes: SET domain‐containing N‐lysine methyltransferases, disruptor of telomeric silencing 1‐like (DOT1L) with lysine as its substrate, and protein arginine transferases (PRMTs) which result in methylation of arginine (Horiuchi et al., 2013; Zhou et al., 2022). According to the products generated, all PRMTs can be divided into two subtypes: Type I PRMTs, including PRMT1, 3, and 4 that generate asymmetric dimethylarginine; and Type II PRMTs, including PRMT 5 and 7 that generate symmetric dimethylarginine. Among them, monomethylarginine is the common product of both type I and type II PRMTs (Horiuchi et al., 2013). PRMT1 has been proven to be an important factor acting downstream of RANKL signaling in osteoclasts and as a participant in the estrogenic inhibition of RANKL‐induced osteoclastogenesis. During this process, JNK signaling is essential for RANKL‐triggered PRMT1 expression, and NF‐κB subunit p65 is activated by PRMT1 for subsequent osteoclastogenesis (Choi et al., 2018). Furthermore, melatonin has been identified as an inhibitor of RANKL‐induced PRMT1 expression and osteoclastogenesis (Choi et al., 2021). In addition to PRMT1, PRMT5 has also been found to stimulate RANKL‐triggered osteoclastogenesis. Through knockdown or inhibition of PRMT5 by using siRNA or EPZ015666, the demethylation of H3R8 and H4R3 in chemokine C‐X‐C motif ligand 10 (CXCL10) and RSAD2 promoters has been shown to participate in this process (Dong et al., 2017). DOT1L has been proven to be a potential protector against OA, and its deficiency can increase the risk of OA along with aging (Cornelis et al., 2019). The demethylation of H3K79 catalyzed by DOT1L is also involved in the suppression of osteoclast fusion and bone resorption. In this context, the expression of CD9 and MMP9 increases during the inhibition of DOT1L. Moreover, the generation of reactive oxygen species (ROS), as well as cellular autophagy, migration, and adhesion of preosteoclasts have also been found to be affected by DOT1L (Gao & Ge, 2018). EZH2, a member of SET domain‐containing N‐lysine methyltransferases, promotes trimethylation of H3K27 in IRF8 promoter following RANKL stimulation, resulting in the inhibition of IRF8 expression and inauguration of NFATc1‐triggered osteoclast differentiation (Fang et al., 2016). A more specific study was conducted leveraging the use of the EZH2 inhibitor GSK126. The results revealed EZH2's predominant mechanism on osteoclast precursors as the suppression of MafB expression via both H3K27 trimethylation and C/EBPβ‐LIP translation during the early stage of differentiation (Adamik et al., 2020).

Another enzyme family also regulates the status of histone methylation, such as the histone lysine demethylase (KDM) (Khaliq et al., 2021). During osteoclastogenesis, KDM4B is overexpressed in response to the RANKL‐JNK signaling and subsequently promotes differentiation but not the proliferation of osteoclast precursors (Yi et al., 2021). The Jmjc domain in KDM4B is important for its interaction with CCAR1, and the KDM4B‐CCAR1‐MED1 complex localizes at the transcription start sites (TSS) of osteoclastogenic genes, such as Fosl2 and Tpm1, upon the activation of RANKL‐RANK signaling (Vicioso‐Mantis et al., 2022; Yi et al., 2021). The complex‐induced demethylation of H3K9me3 in these genes leads to a shift of chromatin structure into an open status, thereby facilitating NF‐κB p65 binding and ultimately promoting osteoclastogenesis (Yi et al., 2021). Furthermore, increased expression of KDM4B has been observed in the inflammatory milieu, and its inhibition resulted in a suppression of Aa‐LPS‐induced osteoclast formation, indicating a potential role in OA amelioration (Kirkpatrick et al., 2018). Jumonji domain‐containing 3 (Jmjd3) is an H3K27me3 demethylase found at the TSS of NFATc1 upon RANKL stimulation, promoting NFATc1 transcription and subsequent osteoclast differentiation (Yasui et al., 2011). Interestingly, Jmjd3 in osteoblasts was also found to correlate with H3K27me3 demethylation in the EphB4 promoter of osteoblasts, thus executing the function of inhibiting osteoclast formation (Wang, Luo, et al., 2022). Another Jmjd subfamily member, Jmjd7, was identified to decrease during osteoclastogenesis and exert an inhibitory effect during this process (Liu et al., 2018).

#### 3.1.3 Non‐coding RNAs

Although non‐coding RNA does not directly code for mRNA or proteins, recent studies have underscored its significant role in the aging process by regulating gene expression (Earls et al., 2014; Lee & Kang, 2022). Studies have principally focused on alterations in non‐coding RNAs, including microRNAs (miRNAs), long non‐coding RNAs (lncRNAs), and circular RNAs (circRNAs) during aging (Wang, Liu, et al., 2022).

MiRNAs are single‐stranded non‐coding RNAs with approximately 20 nucleotides (nts) in length and regulate gene expression at the post‐transcriptional level (Lu & Rothenberg, 2018). Extensive studies have depicted numerous differentially expressed miRNAs associated with OA, which involve a range of signaling pathways, such as RANKL/RANK/OPG, MAPK, and PI3K/AKT signaling pathways, further regulating the cellular differentiation, proliferation, resorption, and apoptosis of osteoclasts (Duan et al., 2020; Franceschetti et al., 2014; Inoue et al., 2021). Maki Uenaka et al., via the use of an intravital optical imaging system, unveiled that small osteoblast vesicles containing miR‐143‐3p are secreted by and absorbed into osteoblasts. These vesicles can downregulate the expression of OPG while upregulating RANKL expression in osteoblasts, thereby promoting osteoclastogenesis through a cell–cell communication (Uenaka et al., 2022). NF‐κB, an important TF in the RANKL‐RANK signaling pathway, controls the expression of monocyte‐specific genes during osteoclastogenesis. The interaction between the p65 subunit of NF‐κB and TSS of two miRNAs (miR99b/let‐7e/125a and miR‐212/132) leads to the upregulation of these miRNAs. Consequentially, the downregulated genes during osteoclastogenesis, such as TNFAIP3, IGF1R, CX3CR1 and IL‐15, are post‐transcriptionally silenced (de la Rica et al., 2015).

LncRNAs are non‐coding transcripts exceeding 200 nts in length. An integrated analysis uncovered seven differentially expressed hub lncRNAs (LINC00313, LINC00654, LINC00839, TBC1D3P1‐DHX40P1, HCP5, MIR210HG, and ISM1‐AS1) in OA, which were found to be enriched in osteoclast differentiation‐related pathway (Chen & Chen, 2020). During skeletal aging, lncRNA nuclear‐enriched abundant transcript 1 (NEAT1) demonstrated elevated expression levels, promoting the secretion of extracellular vesicles (EVs) containing CSF1 and contributing to osteoclast differentiation (Zhang, Xu, et al., 2022).

CircRNAs, characterized by their single‐strand covalent loop structures, play regulatory roles in various biological processes through interactions with miRNAs (Zheng et al., 2021). In the context of OA, circFNDC3B acts as a defender through binding with miR‐525‐5p to activate heme oxygenase‐1 (HO‐1) and inhibit NF‐κB signaling pathway (Chen, Huang, et al., 2023). Furthermore, exerting as the miRNA sponges, more circRNAs, including circFam190a, circHmbox1, CircRNA_25487, and circRNA_28313 have also demonstrated potential for osteoclast regulation (Chen, Chen, et al., 2023; Chen, Ouyang, et al., 2019; Liu et al., 2020; Zhang et al., 2021).

### 3.2 Maintenance of proteostasis

Proteostasis is a fine coordinated system maintained by three basic pathways: protein synthesis and folding, conformational stability, and protein degradation. The proteostasis networks ensure all correctly folded proteins with proper solubility and functionality are delivered to the right location at the right time. However, maintenance of proteostasis is challenging work due to various internal and external stressors encountered during aging. Proteostasis perturbation is a prominent hallmark of aging and contributes to the pathogenesis of numerous age‐related diseases (Hipp et al., 2019; Kaushik & Cuervo, 2015). Therefore, investigating the association between OA pathology and changes in proteostasis networks is essential.

#### 3.2.1 Molecular chaperones

Molecular chaperones are essential in the proteostasis networks, as they are instrumental in protein synthesis, folding, stabilization and degradation. However, depletion, destabilization, and mutation in chaperones occur during the aging process, leading to protein misfolding and aggregates (Kaushik & Cuervo, 2015). The loss of molecular chaperones along with aging has been shown to disrupt proteostasis and contribute to pathological alterations in bone, cartilage, and stromal tissue during OA (Lambrecht et al., 2014).

Heat shock protein (Hsp) family, the most typical chaperones, is generally synthesized under stress conditions, and its treatment could attenuate bone and cartilage destructions in OA (Chen et al., 2014; Kim et al., 2013). It was first observed in 2005 that the suppression of the N‐terminal ATPase domain of HSP90 enhances osteoclastogenesis (Price et al., 2005). A subsequent study indicated that this HSP90‐inhibition process facilitated osteoclast formation by amplifying and activating the microphthalmia TF (MITF), a downstream effector in RANKL signaling, bypassing NFATc1 dependency (van der Kraan et al., 2013). Ryan C. Chai et al. extended this understanding, showing that Hsp90 inhibitors along with other cell stress agents lead to the disassociation and activation of the heat shock factor (Hsf1) from the HSP90 complex. Moreover, the overexpression of MITF was tied to Hsf1 activation, potentially due to the polymerization and modification of Hsf1, consequent to its binding to the MITF promoter (Chai et al., 2014). Similarly, the formation of the Hsp90‐Cdc37‐AZI2 complex suppresses proto‐oncogene tyrosine‐protein kinase (c‐Src) activation, a direct client protein of Hsp90, thereby negatively affecting osteoclast survival (Maruyama et al., 2015). However, opposing views also exist. Two former studies proposed that inhibition of the ATP‐binding domain of Hsp90 could actually inhibit osteoclast formation via the disruption of ERK and AKT signaling pathways (Huston et al., 2008; Okawa et al., 2009). Furthermore, the interaction between Hsp90 and Rab11b suppresses the transport of c‐fms and RANK receptors presented on the osteoclast surface to lysosomes mediated by Rab11b (Tran et al., 2021).

Protein disulfide isomerase (PDI), primarily situated in the ER, exhibits both molecular chaperone and enzyme activities (Wang, Wang, & Wang, 2015). The aggregation of unfolded proteins, a consequence of proteostasis imbalance in the aging process, triggers the unfolded protein reaction (UPR), leading to ER stress (Santra et al., 2019). Functioning as a molecular chaperone, PDI alleviates UPR in a redox‐dependent manner. Specifically, the electron transfer that mediated by ER oxidoreductin 1 (Ero1) from PDI to molecular oxygen is critical for PDI conformation change. However, this process also generates ROS as a byproduct (Tsai et al., 2001; Tsai & Rapoport, 2002). Excessive oxidative stress further exacerbates ER stress during aging (Liu et al., 2019). In addition, ER stress and UPR can initiate the secretion of inflammatory factors and activate inflammatory reactions (Victor et al., 2021). Therefore, an intimate interplay potentially exists among UPR, ER stress, oxidative stress and inflammation during aging, with PDI playing a central role in this network. Age‐related alterations in PDI have been linked to OA, oral dryness, and the senescence of epithelial, endothelial, and mesenchymal stem cells (Bhattarai et al., 2018; Cheng et al., 2023; Kim, Youn, et al., 2018; O'Sullivan et al., 2022; Tan et al., 2020), whereas the role of PDI in osteoclast lineage has been partially revealed. Through the suppression of ROS and the NF‐κB signaling, PDI inhibition or knockout has been shown to mitigate the inflammatory phenotype of osteoclast precursors (Xiao et al., 2018). Furthermore, our published work highlighted the important role of PDI in osteoclastogenesis by affecting the cellular redox state (Wang, Yuan, et al., 2023). Interestingly, a decrease in the osteoclast number was also observed in a cartilage‐specific PDI knockout model (Linz et al., 2015).

#### 3.2.2 Autophagy

Autophagy is the most important proteolytic system responsible for the degradation and recycling of cellular and extracellular components to balance the need for nutrients and energy. Ample evidence has revealed a strong correlation between autophagy deficiency and the aging process. A collection of highly conserved genes named autophagy‐related genes (Atg) broadly participate in the regulation of the entire autophagy process. Decreased lifespan has been found to correlate with Atg knockdown or mutation, which, in most cases, leads to various degenerative diseases. Conversely, enhanced autophagy also emerges in some short‐lived models (Lapierre et al., 2015). While the specific regulatory mechanisms of autophagy in the aging process have not been fully elucidated, the close association and parallels between autophagy and bone resorption have encouraged further investigation. Similarities include the shared property of substance degradation, the requirement for lysosome components to function, and a strong relationship with aging. Thus, increasing studies are focusing on the link between autophagy and osteoclast activity, ultimately aiming to unveil its involvement in degenerative diseases such as OA.

A reduced number of osteoclasts and suppressed function of bone resorption were found in the Atg7‐knockdown model (Lin et al., 2013). Moreover, the localization and fusion of secretory lysosomes to the ruffled border demonstrated a strong Atg‐dependent pattern, and the bone resorption function was greatly inhibited when Atg5 was knockdown (DeSelm et al., 2011). Through bioinformatics analyses, differentially expressed genes (DEGs) including Becn1, Atg3, and Atg12 were identified in OA. Following histopathological staining further reveals a correlation between increased expression of these genes and osteoclast activation in the subchondral bone of OA patients (Zhang, Sun, et al., 2022).

**FIGURE 3:**
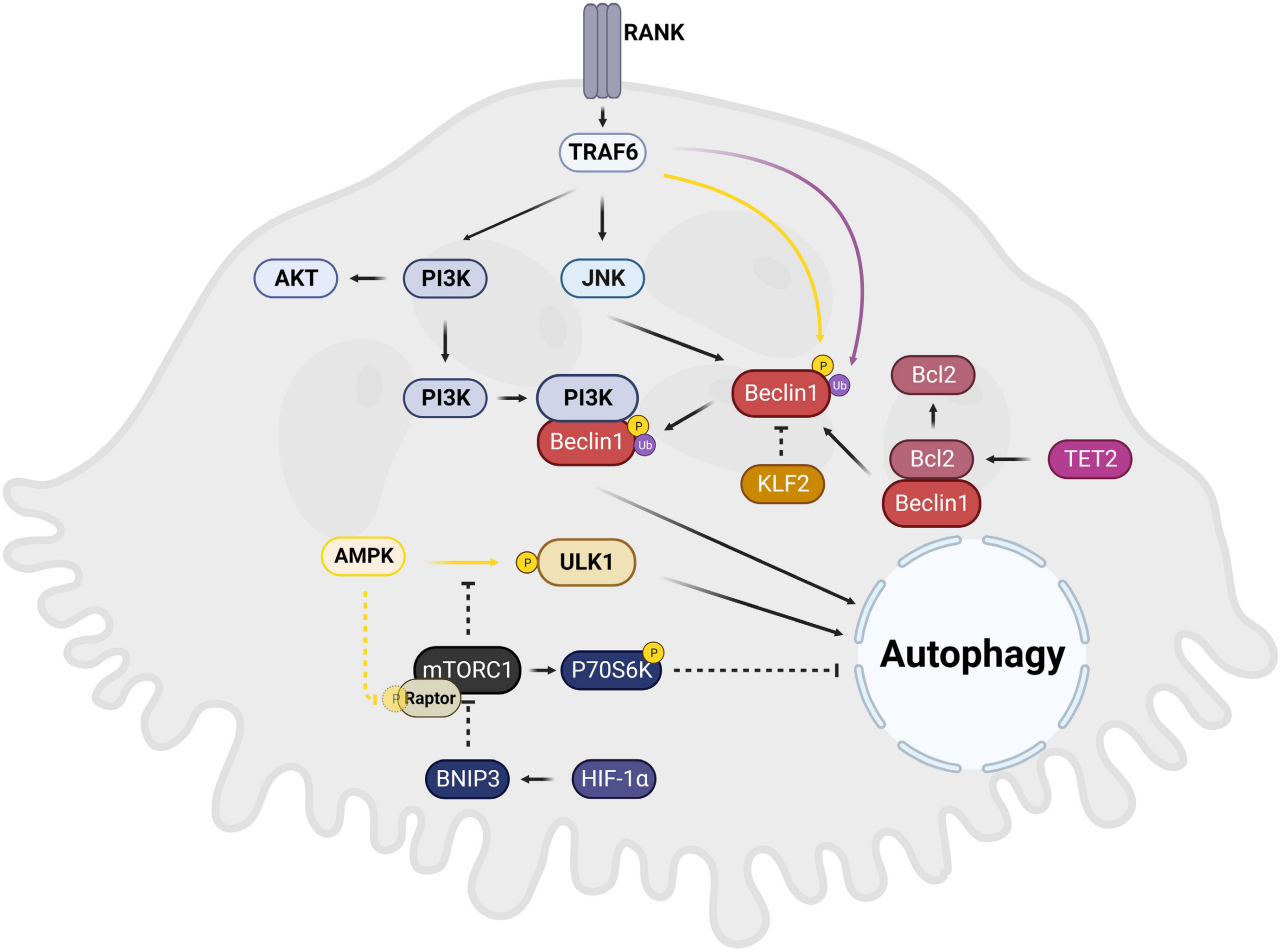
Autophagy‐related signaling transduction in osteoclasts. Three major complexes are involved in the autophagy process: the mTORC1, ULK complex, and PI3K complex. Important signaling pathways related to osteoclast activities, such as the RANKL signaling pathway, MAPK signaling pathway, and AMPK signaling pathway, are regulated by these three complexes. AMPK, adenosine monophosphate (AMP) activated protein kinase; Bcl2, B‐cell lymphoma 2; BNIP3, BCL2 interacting protein 3; HIF‐1α, hypoxia inducible factor 1 subunit alpha; JNK, c‐Jun N‐terminal kinase; KLF2, Kruppel‐like factor 2; mTORC1, mechanistic target of rapamycin complex 1; P70S6K, p70 ribosomal protein S6 kinase; PI3K, phosphoinositide 3‐kinase; RANK, receptor activation of NF‐κB; TET2, ten‐eleven translocation 2; TRAF6, tumor necrosis factor receptor‐associated factor 6; ULK1, Unc‐51‐like kinase 1.

The signaling transduction involved in autophagy activation and regulation is intricate, making it difficult to sort out the relationship between autophagy and osteoclasts at the molecule level. Recent research has focused on the formation and regulation of the three major complexes in the autophagy process, namely mTORC1 complex, ULK complex, and PI3K complex, as well as their regulation via the RANKL signaling pathway, MAPK signaling pathway, and AMPK signaling pathway (Aman et al., 2021; Tong et al., 2022) (Figure 3).

Beclin1 serves as an essential hub within the PI3K complex, and its overexpression promotes autophagy, as well as the formation and resorption capacity of osteoclasts (Lin et al., 2013). However, the binding of Beclin1 to Bcl2 greatly inhibits its function, and this regulatory mechanism is under the control of various factors. In a TET2 knockdown model, as a DNA demethylase, the overexpression of Bcl2 suppressed Beclin1‐dependent autophagy and the bone resorption process (Yang, Tao, et al., 2022). Additionally, the direct epigenetic regulation of Beclin1 by klf2, as mentioned, could also inhibit osteoclastogenesis (Laha et al., 2019). The interaction between Beclin1 and Bcl2 could be disrupted by the phosphorylation of Beclin1 at the Thr119 site via GPCR kinase 2‐interacting protein 1 under starvation condition (Zhao et al., 2018). Aside from the canonical NF‐κB and MAPK signaling pathways, autophagy provides an alternative route for RANKL‐induced osteoclastogenesis. Either phosphorylation of Bcl2 at the Ser70 site prompted by RANKL (Ke et al., 2022) or ubiquitination of Beclin1 at the K117 site mediated by TRPV6 under RANKL stimulation (Arai et al., 2019) will lead to Beclin1‐mediated autophagy. JNK1, a downstream factor in RANKL‐induced osteoclastogenesis, has also been proven to participate in regulating the disassociation of Beclin1 and Bcl2 induced by RANKL (Ke et al., 2019).

An intricate coordinating network exists among the AMPK signaling pathway, mTORC1 complex, and ULK1 complex. AMPK‐mediated phosphorylation of the ULK1 complex promotes its activation, subsequently triggering the PI3K complex and initiating autophagy. The mTORC1 complex serves as a negative regulator in autophagy activation, impeding the interaction between AMPK and ULK1 complex. However, the function of the mTORC1 complex is also regulated by the phosphorylated inhibition from AMPK, which phosphorylates Raptor in the mTORC1 complex (Guo, Su, et al., 2021; Wang, Deng, et al., 2020; Yin et al., 2019). HIF‐1α has been proven to be an upstream activator for BNIP3, which subsequently inhibits the mTORC1 complex and facilitates autophagy. Therefore, the connection between autophagy and hypoxia‐induced osteoclastogenesis has been established (Zhao et al., 2012). In addition, p70S6K has been identified as an inhibitor for autophagy, working downstream of the mTORC1 complex. Activation of the mTOR‐p70S6K signaling pathway by orcinol glucoside suppresses autophagy and osteoclast differentiation (Gong et al., 2022).

Numerous studies have investigated the involvement of chaperones and autophagy in osteoclast formation and function, establishing a close relationship between them and their contribution to OA progression during aging. However, the current literature displays limitations, as most studies focus on the regulatory aspects of autophagy or chaperone that can trigger alterations in the cellular activities of osteoclasts, without elucidating the specific connections between changes in these processes or in osteoclasts. Consequently, more comprehensive studies aiming at the key juncture between autophagy or chaperones and osteoclasts may prove meaningful. Furthermore, considering the importance of chaperones and autophagy in proteostasis maintenance, a proteomic approach will be helpful in thoroughly elucidating their interplay from a holistic aspect.

### 3.3 Cellular senescence

The concept of cellular senescence was first introduced in 1961 and classically described a phenomenon in which cells cease proliferation and irreversibly reach a stable and terminal state (Hayflick & Moorhead, 1961). Considering that osteoclast is a post‐mitotic cell in full differentiation status, this classical phenotype of cell cycle arrest will not be discussed here. Subsequent research has further enriched this concept, exploring pleiotropic senescent phenotypes such as SASP, DNA damage response, apoptosis resistance, morphological alterations, and so on (Hernandez‐Segura et al., 2018). The initial intention of acute senescence is to eliminate damaged cells and facilitate tissue regeneration via the recruitment of macrophages for self‐clearance. Nevertheless, this otherwise beneficial process would become detrimental if the clearance of senescent cells lags behind their generation (Munoz‐Espin & Serrano, 2014).

SASP serves as the primary approach of intercellular communication between senescent cells to either normal cells or other senescent cells. As senescent cells accumulate, continuous overproduction of SASP‐mediated proinflammatory cytokines and proteases will induce an age‐related, chronic, systematic, and spontaneous inflammatory state termed “inflammaging” (Fafian‐Labora & O'Loghlen, 2020; Xie et al., 2021). In the context of inflammatory diseases which affect entire joints, the ability to secrete inflammatory cytokines and chemokines by synovial fibroblasts, immune cells, adipocytes, as well as chondrocytes and other bone cells may be enhanced by senescent cells and oxidative stress because of aging (Coryell et al., 2021). The severity of the local inflammatory condition is highly correlated with OA progression as mentioned. More specifically, injecting senescent cells into the knee joint has been proven to induce characteristic radiographic, histological, and symptomatic alternations of OA in animal models, providing direct evidence of the connection between cellular senescence and OA occurrence (Xu et al., 2017). SASP and the associated inflammaging can be considered the primary driving force of cellular senescence in OA pathology, contributing to the formation of an inflammatory microenvironment within OA joints and strengthening bone resorption. Enhancements in osteoclast formation and function are observed in aging bone and in vitro cultivation, which can be attributed to two aspects: One is the unique characteristic of senescent osteoclasts, while the other is the intercellular communication between surrounding senescent bone cells on osteoclasts, which primarily relies on SASP.

Unlike other cells aging, senescent osteoclasts behave with even greater activity compared to normal conditions. An in vivo study confirmed the expression of classical senescent marks such as p16 and p21 in osteoclasts along with enhanced bone resorption activity (Lee et al., 2021). In addition, the production of MMP9, CTSK, and other SASP factors is also increased in senescent osteoclasts, promoting the bone resorption function (Gorissen et al., 2018). Interestingly, CTSK can work not only as a bone‐resorbing enzyme but also as a senescence‐promoter, affecting the nearby non‐senescent osteoclasts through a paracrine pathway (Chen et al., 2007). Other than stem cell exhaustion and cell cycle arrest generally observed in most aging tissues, the potential for enhanced osteoclastogenesis had been identified to be positively correlated with the aging process. However, its clearance did not successfully inhibit bone resorption function in a mouse model (Kim et al., 2019). SASP was also observed in senescent preosteoclasts which contribute to metabolic syndrome‐associated OA through the cyclooxygenase 2/prostaglandin E2 pathway. However, by using conditioned medium from non‐senescent preosteoclasts and senescent preosteoclasts, the SASP secreted by senescent preosteoclasts inhibited the differentiation capacity of normal osteoclast precursors (Su et al., 2022).

Aside from the senescence of osteoclast itself, it is also well‐established that SASP and other senescent phenotypes can be found in osteocytes, osteoblasts, chondrocytes and synovial fibroblasts in various in vivo and vitro models (Del Rey et al., 2019; Diekman et al., 2018; Farr et al., 2016). The inflammatory microenvironment and the SASP that secreted, such as IL‐1, IL‐6, IL‐17, and tumor necrosis factor‐α (TNF‐α) exhibit a positive regulatory effect on osteoclast differentiation and function (Son et al., 2020; Yao et al., 2021; Yokota et al., 2021; Zhang et al., 2019). An increased expression of Tnfsf11 and overproduction of RANKL were also found in aged osteocytes, providing extra osteoclastogenic factors to osteoclast precursors (Farr et al., 2017). Notably, Joshua N. Farr et al. directly identified the role of senescent cells and SASP in osteoclastogenesis and bone resorption. By using the INK‐ATTAC mouse model or senolytics (AP20187) to eliminate the senescent cells, or using senomorphics (ruxolitinib) to inhibit SASP, the researchers found fewer osteoclast numbers and bone resorption in the experimental groups. The in vitro study using the conditioned medium harvested from senescent cells to culture osteoclast also yielded similar results (Farr et al., 2017).

Therefore, the heightened inflammatory milieu and increased osteoclast activity are prominent features of bone cell senescence in the aging skeleton, consequently resulting in a greater risk of OA in elder individuals. Interventions targeting senescent cells and SASP may potentially alleviate inflammaging and offer therapeutic benefits for OA.

### 3.4 Mitochondrial dysfunction

During aging, the deterioration of mitophagy leads to the accumulation of dysfunctional mitochondria, characteristic by alterations in morphology, oxidative phosphorylation (OXPHOS), and mitochondrial DNA (mtDNA) (Onishi et al., 2021). A major consequence of this mitochondrial dysfunction is the overproduction of reactive oxygen species (ROS), which further results in the extensive loss of function in various electron transport chain complexes within senescent mitochondria (Boengler et al., 2017; Nolfi‐Donegan et al., 2020). ROS is a well‐known promotor of osteoclastogenesis and at the same time drives the progression of OA (Yajun et al., 2021). Moreover, excessive ROS from mitochondria not only induces aging‐related alterations in and outside the cell, but also interferes with mitochondrial homeostasis and damages mtDNA, which further impairs mitochondrial functions including OXPHOS (Yang et al., 2021). Thus, a detrimental positive feedback loop involving excessive ROS production is established. Additionally, a disordered ROS scavenger system also contributes to ROS accumulation and OA pathogenesis in elder individuals (Fu et al., 2016). Nrf2, FOXO, and SIRT act as major regulators of cytoprotective enzymes which are responsible for ROS clearance, but their expressions and activities decrease with age. For example, reduced activity of complex I lowers NAD^+^ concentration, subsequently repressing the activation of FOXO, manganese superoxide dismutase (MnSOD), and catalase via SIRT (van de Ven et al., 2017; Verdin, 2015). Furthermore, the loss of Nrf2‐mediated antioxidant mechanisms during aging enhances osteoclastogenesis and bone resorption, which could be alleviated via the use of Nrf2 activators (Xue et al., 2021).

With further in‐depth research on the association between mitochondrial dysfunction and aging, conflicting opinions emerge, proposing that mitochondrial dysfunction may not be considered as a hallmark of aging. This notion arises from the fact that neither oxidative stress nor mitochondrial mutation is sufficient to solely induce aging‐associated phenotypes, and their attenuation can not always reverse or delay the aging process (Doonan et al., 2008; Kennedy et al., 2013; Vermulst et al., 2007). Conversely, Mclk1^+/−^ mutant mice which display elevated ROS levels, have shown enhanced immunity and extended lifespan (Hekimi, 2013; Yang & Hekimi, 2010). However, compelling evidence also exists that ROS plays an important role in regulating stress‐induced cellular senescence. Meanwhile, shortened telomeres, DNA damage response signaling pathways, as well as SASP have also been proven to be directly related to ROS (Passos et al., 2010; Srinivas et al., 2019; von Zglinicki, 2002).

Undoubtedly, mitochondrial dysfunction increases with aging, accompanied by ROS elevation. Although these endogenous ROS from mitochondria only comprise a small portion of total ROS, their impact on the pathogenesis of OA is significant. In fact, the inflammaging milieu in OA exacerbates local oxidative stress, and the accumulated ROS greatly promotes osteoclast formation and function, primarily through the following two pathways.

**FIGURE 4:**
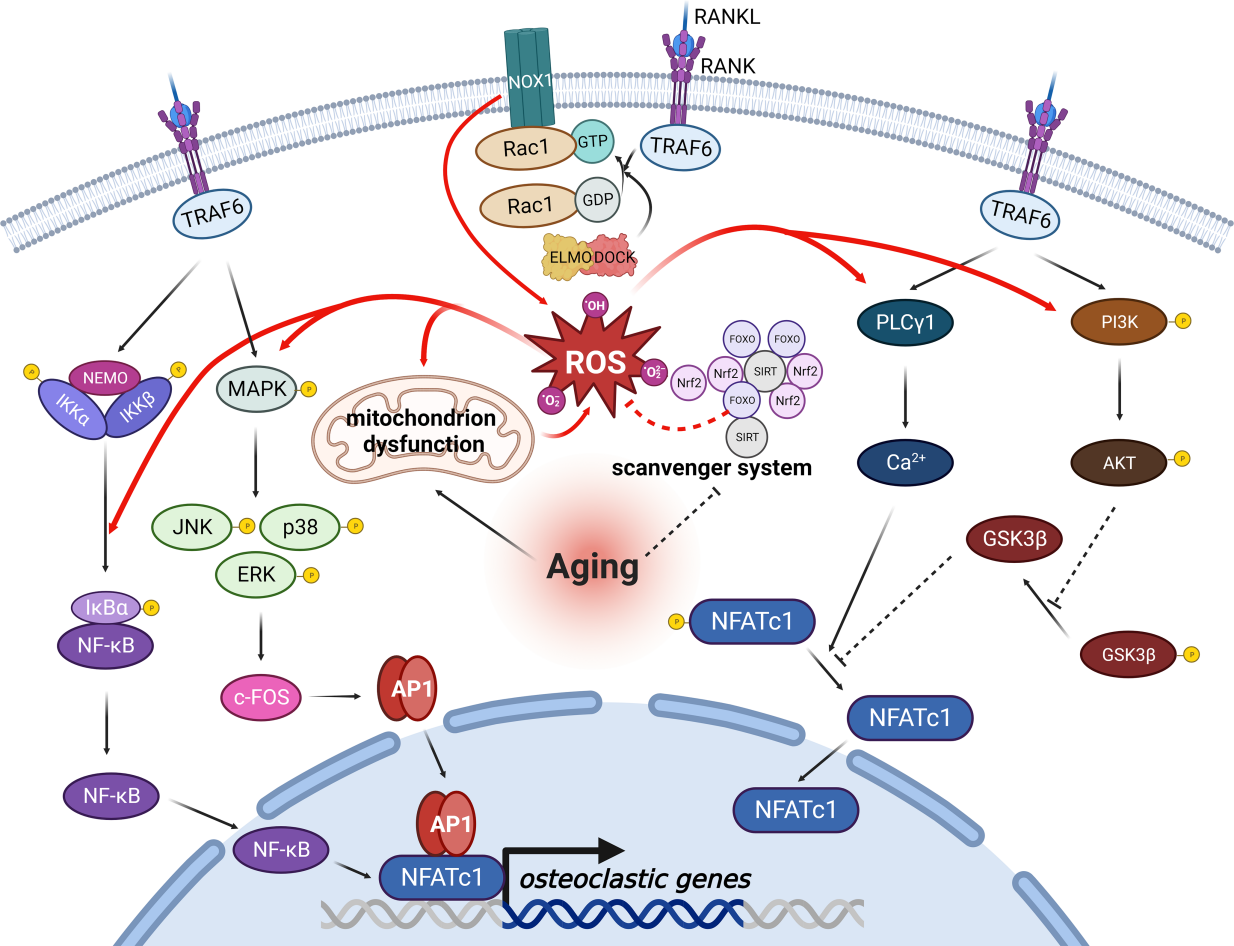
Reactive oxygen species (ROS)‐related signaling pathway in osteoclast. ROS mediate osteoclast activation is closely related to age‐associated alterations. ROS production is indispensable for downstream signaling transduction. However, during the aging process, the generation of ROS is robustly increased due to mitochondrial dysfunction and a disordered scavenger system, thus resulting in the upregulation of osteoclastic gene expression. AP1, activator protein‐1; DOCK, dedicator of cytokinesis; ELMO, engulfment and cell motility; ERK, extracellular regulated MAP kinase; FOXO, forkhead box protein; GDP, guanosine diphosphate; GSK3β, glycogen synthase kinase 3beta; GTP, guanosine triphosphate; IKKα, inhibitor of NF‐κB kinase subunit α; IKKβ, inhibitor of NF‐κB kinase subunit β; IκBα, NF‐kappa‐B inhibitor α; JNK, c‐Jun N‐terminal kinase; MAPK, mitogen‐activated protein kinases; NEMO, NF‐κB essential modulator; NFATc1, nuclear factor of activated T cells 1; NF‐κB, nuclear factor kappa B; NOX1, nicotinamide adenine dinucleotide phosphate oxidase 1; Nrf2, nuclear factor E2‐related factor 2; PI3K, phosphoinositide 3‐kinase; PLCγ1, phospholipase C gamma1; RANK, receptor activation of NF‐κB; RANKL, receptor activation of NF‐κB ligand; SIRT, sirtuin; TRAF6, tumor necrosis factor receptor‐associated factor 6.

First, a low level of ROS is indispensable for signal transduction in osteoclast differentiation, working as a second messenger in the RANKL signaling pathway (Figure 4). Concurrently, ROS production is controlled by RANKL stimulation. The binding of RANKL to RANK recruits TNF receptor‐associated factor 6 (TRAF6), which serves as a communication hub for downstream signal transduction to the cytosolic component of RANK. Rac1, a member of the Rho‐GTPase subfamily, is also a cytosolic domain and activation hub of NADPH (nicotinamide adenine dinucleotide phosphate) oxidase 1 (Nox1). Inhibition or knockdown of Nox1, as well as the mutation of Rac1, suppress RANKL‐mediated ROS production, revealing the crucial roles of Nox1 and its Rac1 subunit in this process, while TRAF6 and GTP‐Rac1 are vital for Nox1 activation (Lee et al., 2005). In addition, the ELMO/DOCK complex has been proven to be critical for Rac1 activation in this process (Liang et al., 2021). However, Rac deletion may not be an advisable approach for OA treatment, as it is also pivotal for the formation of the actin cytoskeleton during osteoclast differentiation (Razzouk et al., 1999; Wang et al., 2008). Although an age‐related increase of osteoclast numbers was successfully inhibited in mice with conditional knockdown of Rac1 and Rac2 in mature osteoclasts, severe osteopetrosis also appears (Croke et al., 2011; Zhu et al., 2016). While the exact target molecule has not yet been identified, compelling evidence shows that ROS participates in the regulation of downstream factors of TRAF6 in the RANKL signaling pathway, including NF‐κB, P38, ERK, JNK, PI3K, AKT, GSK3β, and PLCγ1. These molecules subsequently act on transcription factors, such as NFATc1, c‐Fos, and activator protein‐1 (AP‐1) to promote gene expression related to osteoclastogenesis (Chen, Qiu, et al., 2019; Kim et al., 2010; Liu et al., 2021; Tao et al., 2020; Wei et al., 2022).

The other pathway through which ROS contributes to OA progression involves the intimate association between oxidative stress and chronic inflammation. Elevated ROS is produced via the corporation of dysfunctional mitochondria along with senescent bone cells under an infammaging milieu, as well as diminished antioxidant systems. Senescent osteoclast does not repress its cellular activity as the normal ones (Gorissen et al., 2018; Little‐Letsinger et al., 2022). It is well‐established that the RANKL‐PGC1β signaling pathway increases both the size and quantity of mitochondria in osteoclasts to meet the demand of all energy‐intensive biological processes during osteoclastogenesis and bone resorption (Park‐Min, 2019). However, excessively generated ROS exacerbates local chronic inflammation and induces lipid and protein peroxidation, which concurrently disrupts the metabolism of bone and cartilage (Marchev et al., 2017). Additionally, accumulated advanced oxidation protein products can directly activate RANK and the receptor for advanced glycation end products (RAGE), initiating the RANK‐NOX1‐ROS axis to promote ROS generation and osteoclast formation (Zhuang et al., 2021).

### 3.5 Other hallmarks

Genomic instability, a primary hallmark of aging, involves alternations in both the integrity and stability of nuclear DNA and mtDNA, as well as the subsequent DNA damage response, which could be induced by ROS. Genomic instability may exacerbate other hallmarks, such as cellular senescence, mitochondrial dysfunction, and certain epigenetic changes (Hou et al., 2019). Telomere attrition, another aging hallmark, contributes to genomic instability. Enhanced bone resorption and osteoclastogenesis, as well as increased DNA damage and senescent cells, were observed in telomerase‐deficient mice. These phenomenons were attributed to the proinflammatory microenvironment caused by telomere shortening, potentially indicating a higher risk of OA (Saeed et al., 2011). Altered intercellular communication is another aspect of aging. However, its major alteration during the aging process consists of the proinflammatory factors and other SASP secreted in an inflammaging context, which has already been thoroughly discussed in previous sections (Aguado et al., 2022).

## 4 SENOTHERAPY AND OA

Therapies targeting a pillar of aging could also generate curative effects on others. Given the central role of cellular senescence among other hallmarks of aging and the extensive works conducted in this field, the primary focus of this section will be emphasizing strategies related to cellular senescence (Tchkonia et al., 2021). Meanwhile, therapies aimed at counteracting inflammaging will also be discussed. By aiming to reduce the harmful effects of senescent cells or directly eliminate them, senotherapies can be broadly classified into two categories: senomorphics and senolytics, thus delaying the progression of aging and its related diseases (Table 1). Due to the huge challenges and costs involved in new drug development, current studies of senotherapies mainly focus on identifying the therapeutic effects of natural compounds or existing drugs which have already received Food and Drug Administration (FDA) approval (Chaib et al., 2022).

**TABLE 1:** Promising senotherapies targeting osteoclasts in OA.

Potential therapeutic approach	Type	Effect	Reference
αIL‐6 neutralizing antibody	Senomorphics	Alleviate joint inflammation and delay disease progression	Keller et al. (2022)
Anti‐IL‐20 monoclonal antibody	Senomorphics	Decrease the severity of subchondral bone damage in OA model and repress joint destruction	Hsu et al. (2017)
IL‐1R1 monoclonal antibody (AMG‐108)	Senomorphics	Decrease WOMAC pain score and repress inflammation status in patients	Cohen et al. (2011)
Anti‐CSF‐1R antibody	Senomorphics	Impede IL‐34 effect, ameliorate osteoclastogenesis, bone erosion and joint pain	Toh et al. (2014), Udomsinprasert et al. (2019)
Resveratrol	Senomorphics	Inhibit NF‐κB signaling pathway, increase SIRT1 expression, interfere osteoclast formation and function, impede OA progression	Inchingolo et al. (2022); Shakibaei et al. (2011); Yang, Sun, and Zhang (2022)
Kaempferol	Senomorphics	Inhibit NF‐κB signaling pathway, repress osteoclastic markers and bone resorption, delay OA progression, repress autophagy through inhibit expression of p62/SQSTM1	Kim, Shin, et al. (2018); Lee et al. (2014); Ma et al. (2021)
NBD pepetides	Senomorphics	Inhibit IKK complex formation to repress osteoclast function and attenuate subchondral bone destruction in OA	Liu et al. (2016)
Rapamycin	Senomorphics	Inhibit mTOR signaling pathway, attenuate subchondral bone related markers and postpone OA progression	Kennedy and Lamming (2016); Li, Huang, et al. (2021)
UBX0101	Senolytics	Clear local senescent cells, ameliorate increased osteoclastic‐related markers, subchondral bone remodeling and OA pain	Jeon et al. (2017)
UBX0101 + Navitoclax	Senolytics	Decrease IL‐17 expression, attenuate inflammatory condition, delay OA progression	Faust et al. (2020)
Fisetin	Senolytics	Inhibit osteoclastogenesis and joint inflammatory condition, improve OARSI score	Yamaura et al. (2022)
Quercetin	Senolytics	Inhibit osteoclastogenesis and bone resorption, prevent subchondral bone destruction in OA joint	Niu et al. (2020); Wang, Yan, et al. (2023)
Dasatinib	Senolytics	Repress SRC kinase to clear senescent cells, inhibit osteoclast survival and function, decrease osteoclastic and bone turnover markers	Hickson et al. (2019); Vandyke et al. (2010)
Dasatinib+Quercetin	Senolytics	Improve bone turnover and bone volume in aging‐induced OA	Zhou et al. (2021)
Dasatinib+Quercetin+Navitoclax	Senolytics	Alleviate joint pain of OA without joint destruction amelioration	Gil et al. (2022)
GM‐CSF monoclonal antibody	Anti‐inflammaging agents	Attenuate OA pain and disease progression	Cook et al. (2012)
IL4‐10 fusion protein	Anti‐inflammaging agents	Attenuate joint inflammation and OA progression	Steen‐Louws et al. (2018)
Manganese dioxide	Nanozymes	Ameliorate inflammation‐induced oxidative stress and manage OA progression	Kumar et al. (2019)
siRNA delivered by nanoparticles	Nanozymes	Decrease osteoclast number and prevent subchondral bone loss in OA	Bedingfield et al. (2021)

### 4.1 Senomorphics

The SASP is one of the major pathways through which senescent cells exert local or systemic effects. Therefore, trying to reduce SASP production or attenuate its adverse effects may serve as potential therapeutic strategies (Ansari et al., 2020). Numerous studies have attempted to neutralize proinflammatory cytokines secreted by senescent cells that contribute to OA (Ansari et al., 2020). In real practice, treatment with antibodies against IL‐6, IL‐20, IL‐1 receptor type 1, and CSF‐1R successfully attenuated inflammatory condition and improved joint structure (Cohen et al., 2011; Hsu et al., 2017; Keller et al., 2022; Toh et al., 2014; Udomsinprasert et al., 2019). Targeting pathways related to SASP production and secretion, such as the JAK/STAT, mTOR, and NF‐κB signaling, appear to be promising senomorphic approaches, with some have been employed in osteoclast regulation and OA studies (Lagoumtzi & Chondrogianni, 2021). Resveratrol and kaempferol, functioning as NF‐κB inhibitors, have exhibited the ability to impede osteoclast activities and exert curative effects on OA through regulating SASP‐related pathways (Inchingolo et al., 2022; Lee et al., 2014; Ma et al., 2021; Yang, Sun, & Zhang, 2022). Reductions in osteoclast number and alleviation in subchondral bone loss have also been observed in treatments with NEMO‐binding domain (NBD) peptides, which inhibit IKK and modulate the IKK/NF‐κB pathway (Liu et al., 2016). Moreover, the inhibitory effects on osteoclast activity can be achieved through epigenetic approaches, for example, promoting SIRT1 via resveratrol or inhibiting p62/SQSTM1 to repress autophagy with kaempferol (Kim, Shin, et al., 2018; Shakibaei et al., 2011). Rapamycin, a well‐studied anti‐aging agent that works as an mTOR inhibitor, demonstrated its potential in OA treatment (Kennedy & Lamming, 2016). The application of rapamycin in the ACLT model successfully attenuated related markers of subchondral bone loss, via the detection of micro–computed tomographic (μCT), thus delaying OA progression (Li, Huang, et al., 2021).

Although some studies have identified senomorphics as a potential strategy for OA treatment, their clinical safety it is still discerning. Senomorphics partly suppress the function of senescent cells without completely eliminating them, which request long‐term usage to achieve a sustained effect. Moreover, some SASP may also function under physiological conditions. Therefore, continuous suppression of SASP will lead to off‐target effects of senomorphics and disruption of homeostasis (Lagoumtzi & Chondrogianni, 2021).

### 4.2 Senolytics

Although senescent cells exhibit proapoptotic and proinflammatory properties, cell death and tissue destruction appear to occur beyond senescent cells. This phenomenon may be attributed to senescent cell antiapoptotic pathways (SCAPs). Consequently, drugs or compounds identified to target SCAPs may exert proapoptotic potential to efficiently induce apoptosis in senescent cells (Chaib et al., 2022).

UBX0101 is a small molecule drug exhibiting senolysis properties by decoupling p53 from MDM2 (Cho et al., 2021). Following ACLT, the accumulated senescent cells in the knee joint were effectively eliminated following UBX0101 administration, along with a significant reduction of osteoclastic markers, subchondral bone sclerosis, and OA pain (Jeon et al., 2017). Meanwhile, therapeutic effects have also been shown in aging‐related spontaneous OA, indicating the potential application of senolytic UBX0101 in both aging and trauma‐induced OA (Jeon et al., 2017). However, the outcome for UBX0101 in phase 2 clinical trial was less than satisfactory, as it failed to eliminate senescent cells or alleviate OA symptoms (Chaib et al., 2022). Interestingly, the combination of UBX0101 and navitoclax (ABT263) has been observed to be effective in an animal model, evidenced by decreased IL‐17 expression, attenuated inflammation, and reduced disease progression following post‐traumatic OA (Faust et al., 2020). Recently, more natural and synthetic compounds with senolytic effects have been identified. Quercetin and fisetin, plant‐derived natural flavonoids, have been shown to decrease local inflammatory markers, alleviate subchondral bone destruction and suppress osteoclast activity in OA (Niu et al., 2020; Wang, Yan, et al., 2023; Yamaura et al., 2022). Identified as a tyrosine kinases inhibitor, dasatinib can efficiently repress SRC kinases and other SCAPs thus to clear senescent cells (Hickson et al., 2019). Both in vitro and vivo studies show that osteoclast survival and function were greatly inhibited by dasatinib, as well as decreased bone turnover markers detected by μCT (Vandyke et al., 2010). The combination treatment of dasatinib and quercetin exhibited an even superior therapeutic effect, which improves bone turnover markers and bone volume in the subchondral bone of aging‐induced OA. This effect was not observed in the younger group, suggesting an aging‐specific therapeutic function (Zhou et al., 2021). However, it was noted that oral administration of navitoclax, quercetin, and dasatinib could relieve OA pain but have no effect on attenuating joint destruction in 20 to 22‐month‐old mice with spontaneous OA (Gil et al., 2022).

Currently, numerous senolytics targeting senescent cells are under development. The senescence‐associated β‐galactosidase (SAβ‐gal) serves as a prominent feature of senescent cells and a potential target for clearance (Guerrero et al., 2020). To address this, an encapsulation strategy using a cytotoxic substance coated by galacto‐oligosaccharides was developed to target senescent cells with high SAβ‐gal activity. The encapsulated drug will be released only when the surface gel is digested by galactosidase in senescent cells (Munoz‐Espin et al., 2018). A prodrug designed with galactose‐modified duocarmycin has demonstrated senolytic function recently, selectively eliminating different types of senescent cells (Guerrero et al., 2020). Despite the promising future of senescent cell‐targeted therapies, their current application in OA treatment remains limited.

### 4.3 Inflammaging relief and other approaches

Apart from senomorphics, other approaches targeting imbalanced cytokines in the inflammatory milieu of OA have also proven effective. For example, the utilization of granulocyte‐macrophage colony‐stimulating factor (GM‐CSF) monoclonal antibody or fusion protein of IL‐4 and IL‐10 has successfully attenuated inflammatory conditions and delayed OA progression (Cook et al., 2012; Steen‐Louws et al., 2018).

Nanozymes, featured by high catalytic activity and antioxidant properties, have exhibited advantages in treating chronic inflammatory diseases, thereby becoming a hot spot of recent research (Wang et al., 2021). Both nanoparticle‐based delivery systems and nanozyme‐based antioxidant agents have demonstrated advancements in OA treatment (Kumar et al., 2019). Osteoclasts that exhibited oxidative disturbance in OA appear to be a potential target for redox‐responsive nanomaterials to facilitate subchondral bone remodeling and alleviate bone destruction (Zhang, Hu, et al., 2022). In addition, a small interfering RNA (siRNA) delivery system has also been shown to reduce the quantity of activated osteoclasts and suppress subchondral bone resorption in post‐traumatic OA (Bedingfield et al., 2021).

DAMPs are small molecules involved in inflammasome activation which may enhance SASP function (Chaib et al., 2022). The binding of DAMPs on RAGE, a member of pathogen‐recognition receptors (PRRs), will induce a proinflammatory response and promote OA progression (Millerand et al., 2019). Interaction between advanced glycation end products (AGEs) and RAGEs has been proven to participate in the pathogenesis of chronic inflammatory diseases (Dong et al., 2022). Soluble RAGE (sRAGE), functioning as a decoy receptor for AGEs, can competitively inhibit AGE ligands, pushing sRAGE as a promising biomarker for chronic inflammatory diseases (Maillard‐Lefebvre et al., 2009). Increased AGEs level along with decreased sRAGE have been found in aging populations and transgenic overexpression of the latter could alleviate inflammaging. Although direct evidence regarding the effect of sRAGE supplements in OA treatment is insufficient, the level of sRAGE has been proven to be highly relevant to disease severity (Chayanupatkul & Honsawek, 2010).

## 5 CONCLUSIVE REMARKS AND FUTURE PERSPECTIVES

Research in recent years has clearly depicted osteoclasts as the key controller that regulates bone metabolism and turnover, with the responsibility for bone resorption. Within an aging context, the hyperactivated osteoclasts which attribute to aging‐related alterations such as cellular senescence and local inflammaging, contribute to the subchondral bone destruction and other associated pathological characteristics of the early stage of OA. While this review has shed light on many regulatory mechanisms governing cellular fates by aging, outstanding questions remain. Loss of function, decreased cellular activity, and stem cell exhaustion are commonly observed features during aging (Aman et al., 2021; Lopez‐Otin et al., 2013), but opposite phenotypes are seen in osteoclasts. The specific mechanism driving the aberrant behavior of osteoclasts is yet to be fully revealed. Likewise, the studies investigating the potent effect of other important aging phenotypes such as deregulated nutrient‐sensing and genomic alterations on osteoclast is insufficient. Also, integrating all aging hallmarks has been a challenge for osteoclasts, due to potential synergetic or antagonistic influence.

The role of osteoclasts in the pathogenesis of OA has been well‐established, propelling specific studies targeting osteoclasts for disease alleviation. For example, anti‐osteoporosis agents like alendronate, raloxifene, teriparatide, and CTSK inhibitors (L‐006235 or MIV‐711), typically with validated osteoclast‐inhibitory function, have shown effectiveness in OA treatment (Bei et al., 2020; Hayami et al., 2012; Liang et al., 2023; Lindstrom et al., 2018; Siebelt et al., 2014). Drugs targeting more than just the musculoskeletal system may also present the potential for inhibiting bone resorption and delaying OA progression (Guo, Ding, et al., 2021; Karsdal et al., 2008). In addition, numerous chemical compounds extracted from natural herbs have been identified to effectively attenuate subchondral bone microarchitecture and OA progression through osteoclast regulation (Ding et al., 2023; Yajun et al., 2021). More profound and extensive research on the association and interactions between osteoclasts, other bone cells, and the local microenvironment will be potentially meaningful in unveiling OA pathogenesis as well as identifying cellular and molecular targets for early‐stage OA intervention.

## AUTHOR CONTRIBUTIONS

HJW participated in initial conceptualization and was a major contributor in writing the manuscript. TY and YW designed and made all figures. CXL and DJL helped with literature search. ZQL and SS critically revised the manuscript and made vital suggestions in revision. All authors reviewed and approved the final manuscript.

## FUNDING INFORMATION

This work was supported by National Natural Science Foundation of China (nos. 82100936 and 82272485), Natural Science Foundation of Shandong Province (no. ZR2021QH077) and Taishan Scholar Foundation of Shandong Province (no. tsqnz20221170).

## CONFLICT OF INTEREST STATEMENT

The authors declare that they have no competing interests.

## Data Availability

Data sharing is not applicable to this article as no datasets were generated or analysed during the current study.
